# Take-off speed in jumping mantises depends on body size and a power-limited mechanism

**DOI:** 10.1242/jeb.133728

**Published:** 2016-07-15

**Authors:** G. P. Sutton, M. Doroshenko, D. A. Cullen, M. Burrows

**Affiliations:** 1 School of Biological Sciences, University of Bristol, Bristol BS8 1UG, UK; 2 Department of Zoology, University of Cambridge, Cambridge CB2 3EJ, UK; 3 Zoological Institute, KU Leuven, Leuven BE 3000, Belgium

**Keywords:** Take-off, Catapult mechanisms, Body mass, Direct muscle contraction, Scaling, Mantis

## Abstract

Many insects such as fleas, froghoppers and grasshoppers use a catapult mechanism to jump, and a direct consequence of this is that their take-off velocities are independent of their mass. In contrast, insects such as mantises, caddis flies and bush crickets propel their jumps by direct muscle contractions. What constrains the jumping performance of insects that use this second mechanism? To answer this question, the jumping performance of the mantis *Stagmomantis theophila* was measured through all its developmental stages, from 5 mg first instar nymphs to 1200 mg adults. Older and heavier mantises have longer hind and middle legs and higher take-off velocities than younger and lighter mantises. The length of the propulsive hind and middle legs scaled approximately isometrically with body mass (exponent=0.29 and 0.32, respectively). The front legs, which do not contribute to propulsion, scaled with an exponent of 0.37. Take-off velocity increased with increasing body mass (exponent=0.12). Time to accelerate increased and maximum acceleration decreased, but the measured power that a given mass of jumping muscle produced remained constant throughout all stages. Mathematical models were used to distinguish between three possible limitations to the scaling relationships: first, an energy*-*limited model (which explains catapult jumpers); second, a power-limited model; and third, an acceleration***-***limited model. Only the model limited by muscle power explained the experimental data. Therefore, the two biomechanical mechanisms impose different limitations on jumping: those involving direct muscle contractions (mantises) are constrained by muscle power, whereas those involving catapult mechanisms are constrained by muscle energy.

## Abstract (precis)

**Summary:** Praying mantises, which use direct muscle contractions to generate jumps, have take-off velocities limited by the amount of power (energy/time) the muscles can produce.

## INTRODUCTION

Many insects are powerful jumpers, with the best able to reach take-off velocities as high as 5 m s^−1^ in acceleration times of less than 1 ms (Burrows, 2003, 2006, 2009). Some species can also jump precisely to targets (Brackenbury, 1996; Brackenbury and Wang, 1995; Collett and Paterson, 1991) by controlling the orientation of the body at take-off (Santer et al., 2005; Sutton and Burrows, 2008, 2010) and its rotation in mid-air (Burrows et al., 2015). Across the wide variety of insects, there are just two broad categories of propulsive mechanism for jumping that involve the use of legs. The first uses a catapult mechanism in which energy is stored in cuticular structures and the second uses direct muscle contractions without energy storage.

In the catapult mechanism, used by insects such as grasshoppers, fleas and froghoppers, energy produced by muscle contraction (muscle force×distance) is generated slowly and stored by deforming a cuticular ‘spring’. The spring then recoils rapidly, releasing the stored energy and delivering considerable power (energy/time) to the legs, which propel the insect into the air (Bennet-Clark and Lucey, 1967; Patek et al., 2011). As mass increases, these insects will have a greater amount of available energy but will also have correspondingly larger opposing inertia. An equivalent increase in both available energy and inertia will thus result in the take-off velocity (and thus the maximum jumping height) being independent of mass. This relationship was formulated as ‘Borelli's law’ in the 17th century (Borelli, 1680) and summarised by Bobbert (2013). Another consequence is that the energy available per unit mass, the energy density (energy/mass), will be constant. For example, in the desert locust (a grasshopper), take-off velocity is similar across individual nymphs with masses ranging from 5 to 1000 mg (Katz and Gosline, 1993). After Borelli, it was found that muscles were limited in both the amount of and the rate at which they produce energy (Alexander, 1995; Hill, 1964; Zajac, 1989). Catapults, however, are not limited by the rate of energy production (Bennet-Clark, 1975; Bennet-Clark and Lucey, 1967; Gronenberg, 1996; Patek et al., 2011). Likewise, the energy released in catapult mechanisms is independent of the length of the propulsive legs. Longer legs do affect the rate at which the energy in the spring is translated into kinetic energy, but do not affect the total energy available (Alexander, 1995). Consequently, even in closely related insects of similar size, there is no correlation between the length of the legs and take-off velocity when using a catapult mechanism (Burrows and Sutton, 2008). The take-off velocity of jumps using a catapult mechanism is thus restricted by the energy a given mass of muscle can produce and then store in the spring (Alexander, 1995; Vogel, 2005b).

The second jumping mechanism uses direct contractions of the muscles to move the legs, which act as levers to transmit forces to the ground. This mechanism is found in insects such as mantises (Burrows et al., 2015), bush crickets (Burrows and Morris, 2003), flies (Hammond and O'Shea, 2007; Trimarchi and Schneiderman, 1995; Zumstein et al., 2004) and moths (Burrows and Dorosenko, 2015). The mechanical principles underlying these jumps are similar to those used by humans and other vertebrates (Zajac, 1993; Alexander, 1995). These insects do not use an energy store and are constrained by physiological limits on the rate at which their muscles can contract. The faster a muscle contracts, the less force it will produce (Hill, 1964; Zajac, 1989). This results in a physiological limit on how much power a given mass of muscle can generate (the power density). The experimentally determined maximum power a muscle can produce ranges between 100 and 500 W kg^−1^ in different animals (Askew and Marsh, 2002; Ellington, 1985, Sawicki et al., 2015). Contrast this with the 160,000 W kg^−1^ (Burrows, 2009) of power that some insects using a catapult mechanism can generate. The smaller the insect, the greater the power needed to jump because of the shorter distances and times that are available to accelerate the body (Alexander, 1995; Vogel, 2005a,b). The take-off velocity of jumps using a muscle/lever mechanism might thus be expected to be restricted by the power a given mass of muscle can produce.

Another possibility has been raised by the consideration of acceleration during the jump by an insect (Sutton and Burrows, 2011; Bonsignori et al., 2013). In these two studies, the forces within the joints decreased at approximately the same rate as the moment arms increased, resulting in nearly constant joint torques and, by extension, accelerations during the jump. If constant acceleration is the limiting factor for insect jumps, then take-off velocity would have a quantitatively distinct relationship with body mass from the other two mechanisms. The quantitative relationship between an animal's size and its take-off velocity would be different depending on whether the jump was constrained by energy density, power density or maximal accceleration.

We therefore sought to determine the fundamental constraint on the take-off velocity of jumps generated by direct muscle contraction. This requires the study of an insect that meets two criteria: first, during all developmental stages, the animal must use same basic jumping mechanism as its body mass increases; second, these stages must be isometrically scaled versions of each other – the individual body proportions should not change as the insect ages. The mantis *Stagmomantis theophila* meets both criteria. We measured the body form in all stages, from first instar nymphs with a mass of 5 mg through to 1200 mg adults, and show that they grow isometrically. We then analysed jumping performance, in particular take-off velocity in the same insects. We compare this result with the jumping performance of similarly sized (5–1000 mg) grasshopper nymphs (Katz and Gosline, 1993), which use a catapult mechanism and for which the key constraining factor is the energy generated by the muscles. Thus, similarly sized mantises and grasshoppers obey different scaling laws in their jumping performance, which are directly attributable to the differing underlying biomechanics.

## MATERIALS AND METHODS

*Stagmomantis theophila* Rehn 1904 (order Mantodea, family Mantidae) were raised in individual containers. Males went through seven nymphal instars before reaching adulthood, whereas females had an additional eighth instar. The jumping performance of 50 mantises were measured and analysed: six each of instars 1–6, five for instar 7, three females for instar 8, and three adult females and three adult males. Sequential images of three jumps by each of these mantises were captured at rates of 1000 s^−1^ and an exposure time of 0.2 ms with a single Photron Fastcam SA3 camera (Photron Europe, West Wycombe, Bucks., UK) fitted with a 100 mm macro Tokina lens. The images had a resolution of 1024×1024 pixels and were fed directly to a computer for later analysis. Jumps were made to a target from a platform made of high density white foam (Plastazote, Watkins and Doncaster, Cranbrook, Kent, UK) 85 mm deep and 150 mm long against a white surrounding background. The target was a 4 mm diameter, 150 mm long, black rod held vertically against a white background. If the target was placed close the mantis it would merely reach out and grab it and if too far away it would not jump at all. For each instar, the target was moved to the furthest distance away from the platform to which a mantis would jump. This maximal distance for eliciting jumps depended on the age and hence size of the mantis: for a sixth instar mantis the target was 60–80 mm (1.5 to 2 body lengths) from the edge of the platform and for other ages the target distance was related to body size. All the jumps were volitional. It is unknown whether they represent the furthest the mantises were physically able to jump, or the furthest they were willing to jump under this laboratory setting. Selected image files were analysed with Motionscope camera software (Redlake Imaging, Tucson, AZ, USA) or with Canvas 14 (ACD Systems International, Seattle, WA, USA). Take-off was defined as the time at which the last propulsive leg lost contact with the platform and the mantis became airborne. The acceleration time of a jump was defined as the period from the first detectable movement of the propulsive legs until take-off. Peak velocity was calculated as the velocity during a rolling three-point average just before take-off. Temperatures for all experiments ranged from 25 to 30°C. The lengths of the three pairs of legs and of the body of 44 individual mantises of all stages were measured: five each of instars 1–7, three females for instar 8, and three adult females and three adult males. Kinematic and morphometric measurements are given as means±s.e.m.

### Mathematical models and statistics

To estimate the scaling factor of the length of a hind, middle or front leg to body mass, the log_10_ of the leg length (mm) was plotted against the log_10_ of the mass (mg). A linear regression was then performed with Microsoft Excel to find the slope and *R*^2^ values.

Three mathematical models were constructed to predict the relationship between mass (*m*) and take-off velocity (*V*) under the conditions of constant energy, constant acceleration and constant power. Each of these three models provides a simple predicted relationship between the proposed quantity and take-off velocity.

### Constant energy model (Borelli's law)

Here the energy available for jumping is equal to the energy density of muscle (energy per unit mass, β) multiplied by the mass of jumping muscle:
(1)
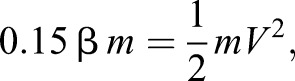


where β is the energy density of muscle, *m* is body mass and *V* is velocity. This equals the kinetic energy at take-off. For all instars and adults, we assumed that the percentage of body mass devoted to jumping was 15% (Bennet-Clark, 1975). Changing the percentage of body mass devoted to jumping muscle does affect the intercept of the models, but does not affect the slope. Our data analysis depended only upon the slopes, and not the intercepts, thus changing this assumption by ±10% did not quantitatively affect any of our conclusions.

This equation can then be solved for the velocity at take-off:
(2)
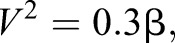

(3)
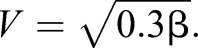


Velocity is constant with respect to mass, predicting a 0 slope on the regression. Eqn 1 shows that absolute velocity is proportional to the normalized energy (energy/mass), meaning that take-off velocity is effectively a normalised variable. Eqn 1 also shows that the energy density (energy/mass) of the jump is proportional to the square of the take-off velocity. Consequently, take-off velocity can be used as a proxy for energy density.

The constant energy model reflected a limit on the energy available for jumping. Predicted take-off velocities were derived by setting the energy density to achieve the mean take-off velocity across all mantises of 0.89±0.19 m s^−1^ (*N*=50) measured from the kinematics.

### Constant acceleration

Here the velocity at take-off is the acceleration multiplied by acceleration time of the jump:
(4)
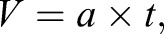


where *a* is acceleration (m s^−2^) and *t* is acceleration time (s).

Eqn 4 can then be integrated to calculate the take-off time in terms of the acceleration distance (*x*):
(5)
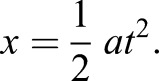


In jumping insects, the acceleration is approximately constant (Bonsignori et al., 2013; Sutton and Burrows, 2011), which allows Eqn 6 to be solved for the take-off time:
(6)
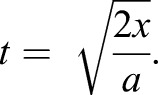


This can then put into Eqn 4 to result in the velocity as a function of acceleration distance:
(7)
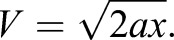


**Fig. 1.**
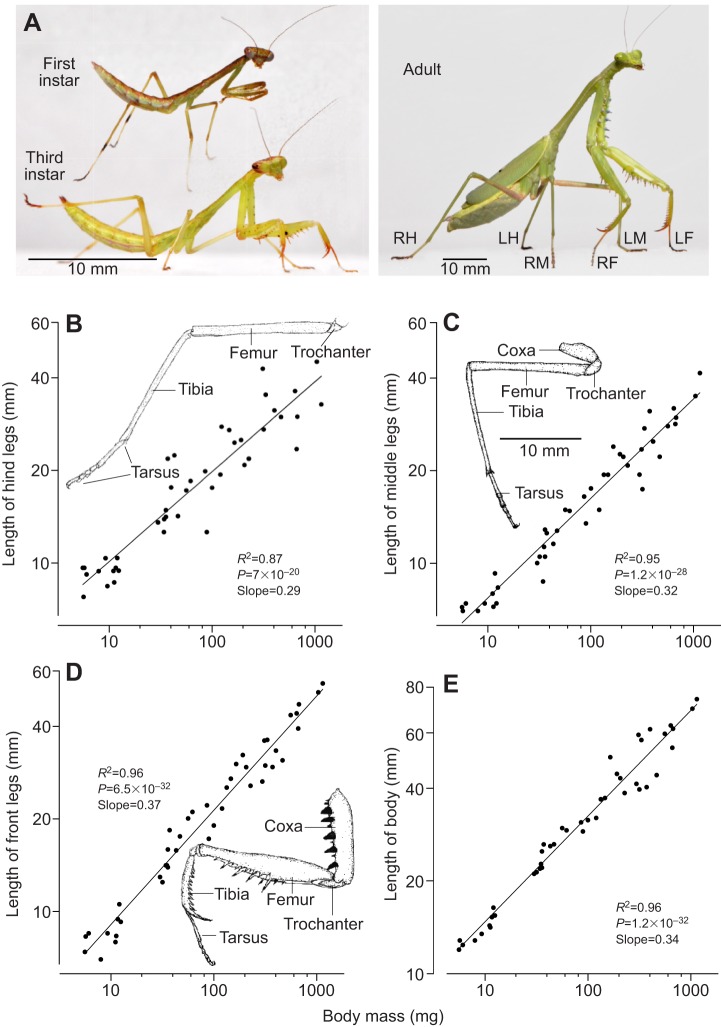
**Morphometry of mantises as related to jumping.** (A) Photographs of a first instar nymph, a third instar nymph and an adult female *Stagmomantis theophila*. The legs of the adult are labelled: RH, LH, right and left hind legs; RM, LM, right and left middle legs; RF, LF, right and left front legs. (B) The length of the hind legs scales with an exponent of 0.29 over three orders of magnitude of body mass. (C) The length of the middle legs scales with an exponent of 0.32 over the same range of body mass. (D) The length of the front legs (which are not involved in jumping) scales with body mass with an exponent of 0.37. Insets show drawings of the three legs. (E) The length of the body scales with body mass with an exponent of 0.34.

If leg length is assumed to scale isometrically with body mass as our experimental results demonstrate (see Fig. 1), the acceleration distance will scale with the cube root of *m* (body mass), which then can be inserted into Eqn 7 to yield:
(8)
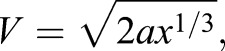
which can be simplified to:
(9)
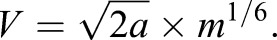

The constant acceleration model reflected a limit on the inertial forces sustainable by the insect. In fleas and leafhoppers, their morphology may keep acceleration constant before take-off (Sutton and Burrows, 2011; Bonsignori et al., 2013), and this model, which predicts a slope of 1/6, reflects that possible constraint. Take-off velocity was estimated by setting the acceleration at the mean value of the average acceleration for all observed jumps, 29.8±6.2 m s^−2^ (*N*=50).

### Constant power

Here the net energy at take-off is equal to the ratio of power divided by mass [the power density (*P*)], multiplied by the mass (*m*) and time (*t*):
(10)
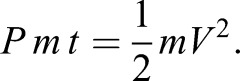


Power density was chosen as a variable (instead of power) because it remains approximately constant across different animals (Zajac, 1989).

The velocity can then be expressed as:
(11)
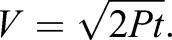
The distance over which this acts (*x*) can then be evaluated by integrating Eqn 10:
(12)
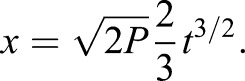
This equation is then solved for *t*:
(13)
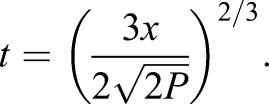


The acceleration distance (*x*) will scale with the cube root of mass. This can be substituted into Eqn 13 to result in:
(14)
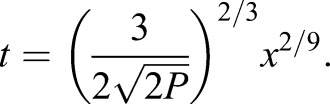
Eqn 14 can then be substituted into Eqn 11 to produce:
(15)
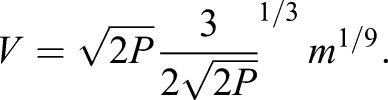


The constant power model reflected a limit on the power available that could be generated by the direct contraction of the jumping muscles. Take-off velocity was estimated by setting the power density (power/mass) at the mean value across all mantises of 87.2 W kg^−1^ of jumping muscle (*N*=50) measured from the kinematics. This model predicts a slope of 1/9.

In all of the above equations, the kinetic energy of the jump (1/2*mV*^2^) is proportional to mass, allowing velocity (without normalising it by mass) to be used as a proxy for the energy density of the animal during a jump.

## RESULTS

### Leg and body lengths indicate that mantises grow isometrically

If take-off velocity is constrained by the energy that a muscle produces, as in catapult jumping mechanisms, it should not be affected by the length of the propulsive legs (Alexander, 1995). By contrast, if take-off velocity is constrained either by muscle power or by acceleration, the length of the propulsive legs should have an effect. We therefore measured the lengths of the three pairs of legs and the body of mantises at all stages in their development.

**Fig. 2.**
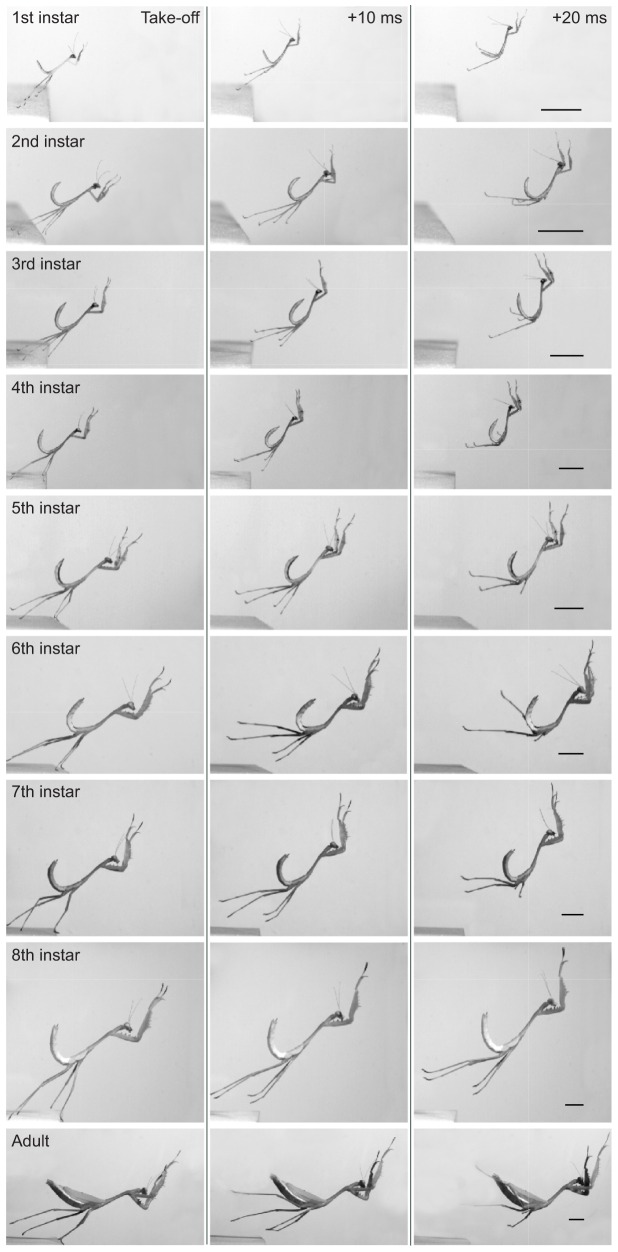
**High-speed images of the kinematics of jumps by mantises.** Jumps, captured at 1000 frames s^−1^, by female first to eighth instars and an adult are shown. For each stage, a frame is shown at take-off and then at 10 and 20 ms after take-off. The movements executed by the legs and the body are similar in all stages. Scale bars: 10 mm.

In a first instar mantis nymph with a mass of 5 mg the length of a hind leg was 11.8±0.1 mm (*N*=6), but in an adult female with a mass of 1200 mg the length was more than three times greater at 37.4±3.5 mm (*N*=3). As mantises grew across all developmental stages (Fig. 1A), the lengths of the hind and middle legs, which generate jumping, both scaled isometrically with body mass: hind legs with an exponent of 0.29 (*R*^2^=0.87, *P*=7×10^−20^, *F*=533, *N*=43; Fig. 1B), middle legs with an exponent of 0.32 (*R*^2^=0.95, *P*=1.2×10^−28^, *F*=772, *N*=43; Fig. 1C). The front legs, which are not directly involved in generating thrust during a jump, also scaled approximately isometrically with body mass with an exponent of 0.37 (*R*^2^=0.96, *P*=6.5×10^−32^, *F*=1119, *N*=43; Fig. 1D). The length of the body also scaled isometrically with body mass with an exponent of 0.34 (*R*^2^=0.95, *P*=1.2×10^−32^, *F*=1120, *N*=43; Fig. 1E). The isometry of the propulsive legs and of the body can be seen in jumps of female mantises of all eight instars and an adult as the mass increased (Fig. 2, Movie 1). From images such as these taken from jumps of all different stages, we could then measure the jumping performance and assess how this was related to body mass during development.

### Jump take-off velocity increases as mantises get larger

**Fig. 3.**
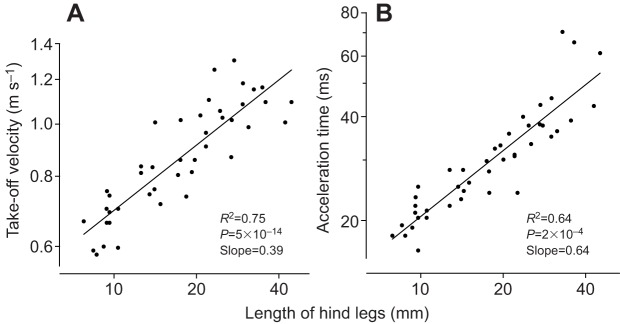
**Jump dynamics scale with leg length.** (A) Mantises with longer legs have higher take-off velocities. (B) Mantises with longer legs have longer acceleration times to take-off. Data for all three log–log graphs are taken from 43 mantises from first instar to adult.

Across all stages, take-off velocity scaled with the length of the hind legs with an exponent of 0.39 (*R*^2^=0.75, *P*=5.4×10^−14^, *F*=124, *N*=43; Fig. 3A). Acceleration times (measured from the first movements of the propulsive legs until take-off) also increased from 20.7±1.0 ms in first instars to 65.9±2.7 ms in adult females, scaling across all stages with an exponent of 0.64 (*R*^2^=0.64, *P*=2×10^−4^, *F*=17, *N*=43; Fig. 3B). Mantises with longer legs therefore had higher take-off velocities. They also had longer acceleration times, because longer legs take more time to be moved in their propulsive jumping movements. The non-zero slope of these correlations suggests that take-off velocity in mantises is constrained by factors different from those that operate in insects using a catapult mechanism.

### Kinematics indicate that muscle power constrains take-off velocity

**Fig. 4.**
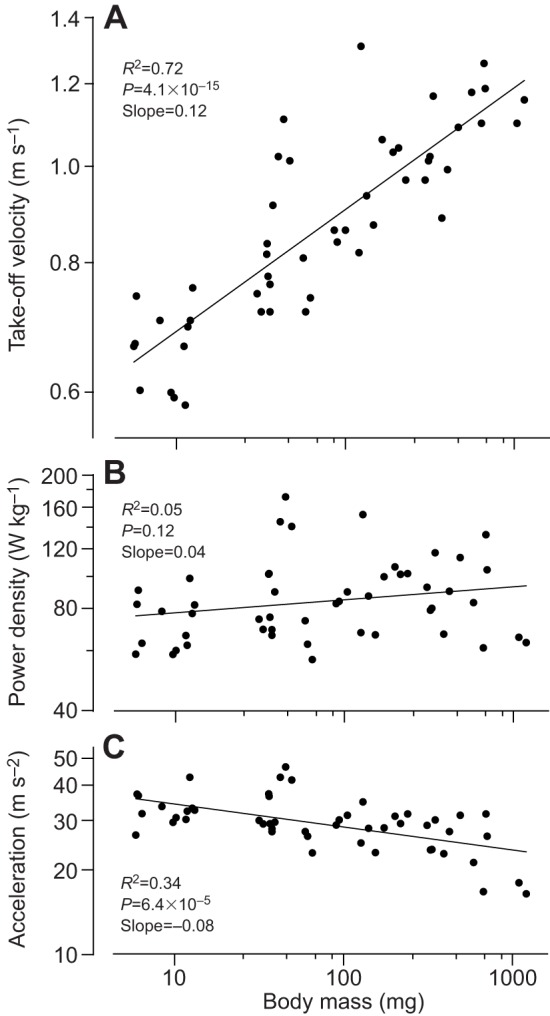
**Jump dynamics of mantises.** (A) Take-off velocity increases with increasing body mass. The points are normally distributed; Shapiro–Wilk test *P*=0.054. (B) Power density stays constant in all mantises, although body mass increases by three orders of magnitude. The points are normally distributed; Shapiro–Wilk test *P*=0.164. (C) Average acceleration before take-off decreases with increasing body mass; the points are normally distributed; Shapiro–Wilk test *P*=0.728. Data for all three log–log plots are the means of three jumps performed by each of 50 mantises from first to seventh instar males and first to eighth instar females, and from adults.

The measured take-off velocity of mantises with larger masses was higher (mean 1.09±0.07 m s^−1^ in adults) compared with those with smaller masses (mean 0.66±0.02 m s^−1^ in first instars). Across all stages, velocity scaled with body mass with an exponent of 0.12 (*R*^2^=0.72, *P*=4.1×10^−15^, *F*=128, *N*=50; Fig. 4A, Table 1). Power density (measured from the kinematics of jumping and based on an estimate that muscles powering jumping make up 15% of body mass) was not significantly different for larger and smaller mantises (*R*^2^=0.05, *P*=0.12, *F*=2.4, *N*=50; Fig. 4B). For example, the mean power density was 68.0 W kg^−1^ in first instars (*N*=6), 69.0 W kg^−1^ in fifth instars (*N*=5) and 63.4 W kg^−1^ in adult females (*N*=3). There were four fourth instar individuals with values over 110 W kg^−1^ (included in Fig. 4B) so that the average power density for all stages was 87.2±25.9 W kg^−1^ (Table 1). Acceleration decreased significantly with increasing body mass with an exponent of −0.08 (*R*^2^=0.34, *P*=6.4×10^−5^, *F*=25, *N*=50; Fig. 4C). For example, acceleration was 32 m s^−2^ in first instars (*N*=6) but fell to 17 m s^−2^ in adult males (*N*=6). Therefore, both velocity and acceleration changed with respect to body mass, but the power density of the muscles was constant. This suggests that power density is the constraining factor on take-off velocity. Detailed kinematic data, including take-off velocity normalised to body length for the jumps by all instars and adults, are given in Table 1.

**Table 1.**
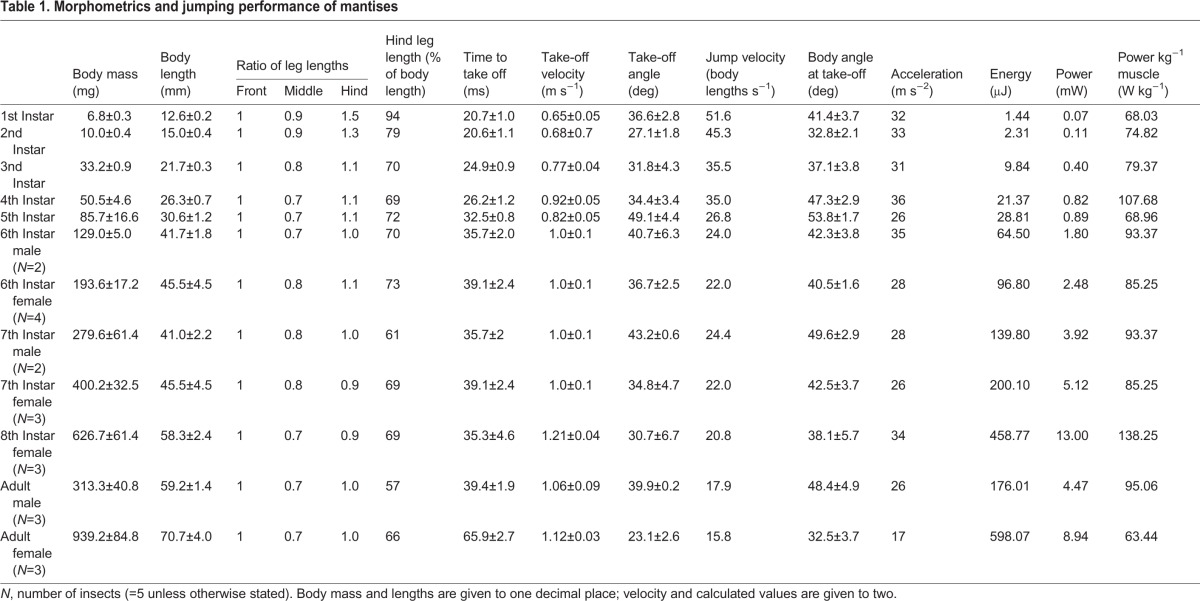
**Morphometrics and jumping performance of mantises**

### A power-limited muscle model best predicts the measured results

To test further for the factors constraining the performance of these jumps, three theoretical, mechanical models (see Materials and methods for derivation) were tested against the measured scaling relationships: (1) a constant energy model (limited by muscle energy, Borelli's law); (2) a constant acceleration model (limited by structural strength of the body); and (3) a constant power model (limited by muscle power).

**Fig. 5.**
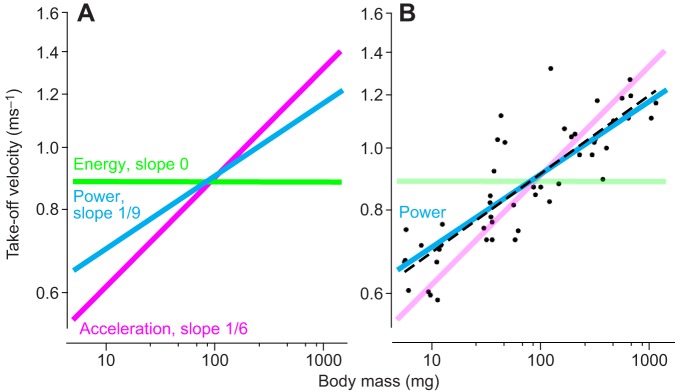
**Model predictions.** (A) Take-off velocity of mantises of different mass in which power (cyan line), acceleration (pink) and energy (green) were independently constrained. (B) Only the model in which power was constrained (cyan line) accurately fitted the experimental data and their regression line (dashed). The other two models tested are indicated by the paler lines.

The constant energy model predicted that take-off velocities should be similar for all body masses (Fig. 5A). However, the prediction (Fig. 5A) from this model was statistically significantly different (*P*=4.1×10^−15^, *F*=128) from the measured data (Fig. 4A).

The constant acceleration model predicted that take-off velocity (*V*) and mass should scale with an exponent of 1/6 (see Eqn 9 in the Materials and methods). The free parameter in this model, acceleration (*a*), only affects the intercept of the line; it does not affect the predicted slope of 1/6. This represents a constraint on the maximum tolerable acceleration by the body during a jump. The prediction from this model (Fig. 5A) was significantly different (*P*=6.4×10^−5^, *F*=19, *N*=50) from the measured data (Fig. 4C). Therefore, this model, which constrains acceleration, did not fit the measured data.

The constant power model predicted that take-off velocities and body mass should scale with an exponent of 1/9 or 0.11 (Fig. 5A; see Eqn 15 in the Materials and methods). As in the acceleration model, the free parameter, power density (*P*), affects the intercept of the line but does not affect the predicted slope. The predicted slope (0.11) from this model (Fig. 5A) was not significantly different from the observed slope of the measured data (0.12) (*P*=0.40, *F*=0.7, *N*=50; Fig. 5B). A model limited by muscle power thus predicted the measured take-off velocities.

## DISCUSSION

Measurements of the body and leg structure of mantises and of their performance in natural jumping show that three specific changes occur as they increase in mass from first instar nymphs to adults. First, the length of the propulsive hind and middle legs scaled with body mass to the power of 0.29 and 0.32, respectively, so that heavier adults had hind legs that were three times longer than those of the smaller and lighter first instar nymphs. Second, the acceleration time to take-off increased more than three times from 20.7 ms in first instars to 65.9 ms in adults. Third, the take-off velocity increased from 0.66 m s^−1^ in first instars to 1.08 m s^−1^ in adults. The power density of the jumping muscles, however, remained the same at 87.2 W kg^−1^ through all developmental stages and into adulthood. The higher take-off velocities achieved by the larger mantises compared with the smaller ones resulted from similar amounts of muscle power that were applied over increasingly longer acceleration times. The conclusion from these measurements is that the take-off velocities of natural jumping are limited by the ability of the muscles to generate power. This is a consequence of the propulsive legs acting as levers controlled by the direct contractions of their muscles. To test further whether muscle power is the underlying limitation to performance, jumping was modelled and three possible factors – power, acceleration and energy – were changed separately. The only model that matched the experimental data was the one in which muscle power was the limiting factor. The model in which acceleration remained constant predicted take-off velocities that increased with body mass, but the exponent of this increase differed significantly from the experimental measurements. Take-off velocity is therefore not constrained by a limitation on the inertial forces (mass×acceleration) that the body can withstand. Similarly, the model in which energy remained constant predicted take-off velocities that would be constant for mantises of different masses. This is also the prediction derived from Borelli's law, and clearly does not reflect the experimental data for mantises, which showed that take-off velocity was proportional to body mass. For the mantis, there is therefore good agreement between the predictions of a power-constrained model and the experimentally determined correlation between body mass and take-off velocity. Both indicate that the primary constraint on take-off velocity is the power generated by the muscles. This conclusion contrasts with that from insects such as grasshopper nymphs, which use a catapult mechanism to jump, even though they have a similar increase in body mass as mantises throughout their larval stages (Katz and Gosline, 1993). Take-off velocity for grasshopper nymphs is independent of body mass (Katz and Gosline, 1993), consistent with limitations on the energy available. Adult grasshoppers have a take-off velocity that is twice that of the nymphs, but this is a reflection of a 50% relative increase in the mass of their jumping muscles and commensurate differences in the morphology of their energy storage device (Gabriel, 1985a,b; Katz and Gosline, 1993).

In all jumping studies, the question arises as to whether the jumps observed represent the maximal performance. This is hard to assess, but it is known that some animals often jump better outside the laboratory (Astley et al., 2013). The scaling rules we have analysed derive from volitional jumps of the mantises to a target. These jumps obeyed a scaling law consistent with physiological limits of power production within muscle. Likewise, the same experimental limitations also apply to the jumping of grasshoppers (Katz and Gosline, 1993), which obeyed a scaling law consistent with the physiological limits of energy production within muscle. Because both studies are of jumps that were volitional, it is appropriate to compare data from the two.

### Effect of leg length on jumping performance

As mantises develop, their propulsive legs grow progressively longer (Fig. 1B) and this increase is correlated with higher take-off velocities. When jumping, the longer legs provide greater leverage and also enable the muscles to contract for longer times, leading to faster take-off velocities. By contrast, in insects that use catapult mechanisms to jump – for example, leafhoppers (Burrows and Sutton, 2008) and grasshopper nymphs (Katz and Gosline, 1993) – longer propulsive legs do not lead to faster take-off velocities, although they are associated with longer acceleration times. A comparison of different species of leafhoppers with similar masses showed that those with longer legs have similar, or even lower, take-off velocities, but have acceleration times that are three times longer than those of short-legged species (Burrows and Sutton, 2008).

Why then do some insects that use a catapult mechanism have longer legs if leg length has no effect on take-off velocity? Speed of take-off may not be the only adaptive value of a jump. Longer legs take longer to be accelerated, whether they are propelled by direct muscle contractions or by a catapult mechanism, and thus the forces exerted on the ground will be spread over a longer time. In turn, this will reduce the energy lost to deformation of compliant surfaces such as leaves. For example, consider two species of leafhopper with similar masses and with similar take-off velocities jumping from the same leaf. The short-legged *Cephalelus angustatus* has propulsive hind legs that are only 20% of body length, but those of the long-legged *Cicadella viridis* are 93% of body length. At take-off, the short-legged *Cephalelus* would lose 66% of its available energy to bending a leaf, whereas the long-legged *Cicadella* would lose only 9% (Burrows and Sutton, 2008). Long legs are therefore clearly advantageous in achieving a higher take-off velocity when jumping from compliant surfaces. Long legs do, however, require more structural reinforcement than shorter legs. The maximum bending moment on a leg is independent of its length, but the compressive forces are inversely proportional to length (Bennet-Clark, 1990) and the tendency to buckle is proportional to the square of the length (Popov, 1990). Thus, despite lower compressive stresses and similar bending stresses, longer legs will have to be more reinforced against buckling (Dirks et al., 2013). The tibiae of some bush crickets with hind legs three times the length of the body will sometimes buckle under the stresses of take-off (M.B., personal observations) and the tibiae of locusts have an inbuilt shock absorber to lessen damage to joints should a hind leg slip at take-off (Bayley et al., 2012).

### Other effects on take-off velocity

As body size increases, energy losses that are due to leg length or wind resistance are likely to alter take-off velocity (Alexander, 1995; Bennet-Clark and Alder, 1979; Scholz et al., 2006). These energy losses should have only a small effect on the take-off velocity of mantises. For example, the potential energy lost to gravity would have its greatest effect on the largest mantises, but would reduce their take-off velocity by less than 1% (Scholz et al., 2006). Likewise, over the 20–60 ms long acceleration phase of the mantis jump, wind resistance would reduce take-off velocity by less than 5% (Bennet-Clark and Alder, 1979). The agreement between the prediction of a power constrained model and the correlation between body mass and take-off velocity thus indicates that the primary constraint on the take-off velocity is the amount of power generated by the muscles. Once airborne, however, wind resistance would reduce jump distance depending on the size and mass of the insect (Bennet-Clark and Alder, 1979; Snelling et al., 2013; Vogel, 2005b).

Many scaling laws are often analysed in terms of behaviour, such as the morphology of the legs and body and the velocity of locomotion (Biewener, 1989; Hooper, 2012; Usherwood, 2013). Mantises and grasshoppers are an example of insects of similar size and mass that engage in the same behaviour – jumping. The biomechanics underlying these movements are, however, different. In mantises, take-off velocity is constrained by the power that can be generated by the direct contraction of muscle. In contrast, the take-off velocity of a grasshopper is constrained by the energy that the muscles can store in the spring of a catapult mechanism. Thus, in these two groups of insects, their differing biomechanics result in the same behaviour being subjected to different scaling laws.
